# Stigma in Multiple Sclerosis: The Important Role of Sense of Coherence and Its Relation to Quality of Life

**DOI:** 10.1007/s12529-021-10030-0

**Published:** 2021-10-12

**Authors:** Lydia Grothe, Matthias Grothe, Judith Wingert, Georg Schomerus, Sven Speerforck

**Affiliations:** 1 Department of Neurology, University Medicine of Greifswald, Greifswald, Germany; 2 Department of Psychiatry, University of Leipzig, Leipzig, Germany

**Keywords:** Stigma, Multiple sclerosis, Quality of life, Sense of coherence

## Abstract

**Background:**

Anticipated and experienced stigma constitute important issues for patients with multiple sclerosis receiving adequate healthcare. Stigma is likely to be associated with lower quality of life in patients with multiple sclerosis, but the underlying mechanisms and contributing factors are unclear.

**Methods:**

We conducted a cross-sectional survey among N = 101 patients with a diagnosis of multiple sclerosis in a German outpatient department. Patients completed questionnaires on enacted and self-stigma (SSCI-8), sense of coherence (SOC-L9) and quality of life (MusiQol). Age, sex, disease duration, disability or extent of limitations (EDSS), cognition (SDMT), depression (BDI-II) and fatigue (FSMC) were used as covariates in linear regression and mediation models.

**Results:**

57.3% of patients with MS reported having experienced stigmatization due to MS at least once. Fatigue (b = -0.199, p < 0.001), enacted stigmatization experience (b = -0.627, p = 0.010) and sense of coherence (b = 0.654, p < 0.001) were significant predictors for quality of life. The mediation analysis showed a partial mediation of the association between enacted stigma and quality of life by patients’ sense of coherence (direct effect: b = -1.042, t = -4.021, p < 0.001; indirect effect: b = -0.773, CI = -1.351—-0.339. The association of self-stigma with quality of life was fully mediated by sense of coherence (b = -1.579, CI = -2.954—-0.669).

**Conclusion:**

Patients with multiple sclerosis are affected by stigma, which is associated with lower quality of life. Sense of coherence is a potentially important mediator of stigma and represents a promising target to refine existing stigma interventions and improve the quality of life in these patients.

## Introduction

Patients with multiple sclerosis (PwMS) often experience negative prejudice, judgment, and exclusion from society because of their disease [1, 2]. This feeling to *“have (or [be] believed to have) an attribute that marks them as different and leads them to be devalued in the eyes of others“* is conceptualized as stigma [3]. Two different forms of stigmatization can be distinguished – public and self-stigmatization. Labeling and discriminating against persons with allegedly undesirable characteristics due to existing cultural stereotypes and prejudices is called public stigmatization [4]. This public stigma subsequently may lead to self-stigma if those affected recognize prevailing negative stereotypes about their condition and agree with them [2; 5]. Enacted stigma refers to the experience of unfair treatment by others [5]. Stigma therefore is always context-specific and not a fixed attribute or characteristic of a person. It has been shown that the experience of discrimination and social withdrawal due to stigmatization can strongly affect patients’ self-confidence and self-efficacy. Stigma is a strong stressor in the everyday life of patients with psychiatric disorders like schizophrenia [6] and is also known to reduce the quality of life (QoL) in patients with neurological diseases [7]. The few studies that have investigated experiences with stigma in PwMS have confirmed the link between stigma and QoL Anagnostouli et al. [8] revealed that both public and self-stigma are associated with impaired QoL. Broersma et al. [9] included sense of coherence (SoC) as another concept and confirmed a positive correlation between the QoL and the SoC of PwMS. The SoC is an important theoretical concept which is closely linked to stigma and QoL. According to Antonovsky, the SoC serves as an individual psychological resource consisting of three components: comprehensibility (the belief that life is structured and explainable), manageability (the belief that one's own resources are sufficient to cope with future challenges) and meaningfulness (the belief that the challenges are worth striving for) [10]. People with a high SoC are aware of their own resources and are able to resort to them in stressful situations. It has been shown that a high level of SoC is effective in buffering stress. A high SoC is also associated with a better QoL, as patients with a higher SoC are more able to cope with the burden of their disease [11]. In a study by Broersma, a high SoC predicted an increased QoL in PwMS and lower enacted and self-stigmatization [9]. Additionally, Johansson et al. [12] revealed that the SoC is a predictor of depressive symptoms and mood in a cohort of PwMS.

Judging by the complex interactions found in existing literature and theoretical concepts, a more complex relationship between stigma, SoC, and QoL seems conceivable. For example, Świtaj [13] investigated and confirmed the SoC as a mediator between stigmatization experiences and the QoL of patients with mental illnesses. They assumed that the experience of stigmatization reduces patients’ self-esteem, which in turn reduces their SoC. In addition, Lundberg et al. [14] showed that experiences of rejection in patients with mental illnesses are associated with a lower SoC. Due to the stigmatization experiences, patients may perceive increased stress and less support [15], which could eventually negatively influence the SoC [16] and subsequently the QoL.

The aim of this study was to explore stigmatization, SoC and their association with QoL for the first time in a German cohort of PwMS, assuming that stigmatization experiences have a negative impact on the perceived QoL of PwMS, whereas SoC is supposed to have a positive impact on the perceived QoL. Additionally, we investigated whether the SoC mediates the association between stigmatization and QoL in PwMS, with increasing experience of stigmatization leading to a lower SoC, which in turn has a negative effect on the QoL.

## Materials and Methods

All 101 PwMS included in the study fulfilled the criteria of multiple sclerosis according to the 2017 McDonald criteria [17]. Exclusion criteria were current or past neurological conditions other than MS and acute clinical relapse within the previous 3 months. Data was taken during routine consultation and from patient records from April to December 2018. The study was approved by the local Ethics committee and all participants gave their written informed consent.

The patients completed questionnaires in German on stigmatization experiences (Stigma Scale for Chronic Illnesses 8-item version (SSCI-8)) [18], Sense of Coherence (Sense of Coherence Scale (SOC-L9)) [19] and quality of life (Multiple Sclerosis International Quality of Life Questionnaire (MusiQol)) [20]. The SSCI-8 was developed for people suffering from neurological conditions and is a short 8-item version of the Stigma Scale for Chronic Illnesses (SSCI) by Rao et al. [2]. It measures two dimensions of stigma, enacted and internalized, with a total score calculated from these two subscores. Statistical analyses were performed with IBM SPSS Statistics 21 with the significance level set at *p* < 0.05. Once all assumptions of regression testing had been met, ten ordinary multiple regression analyses with the following variables were conducted: age, gender, disease duration, disability or extent of limitations (expanded disability status scale, EDSS) [21], cognition (symbol digit matching task, SDMT) [22], depression (Beck depression inventory, BDI-II) [23], fatigue (Fatigue score for motor and cognition, FSMC) [24], enacted stigma, self-stigma, and SoC. Three mediation analysis were performed using the SPSS macro by Preacher and Hayes [25] with a 95% confidence interval and 10,000 bootstrap samples. To correct for multiple comparisons, we used Bonferroni corrected p-values.

## Results

Sample characteristics are shown in Table 1. Participants were mostly female (n = 74; 71.8%), averaged 46.1 years of age, and had a mean disease duration of 10.72 years. Most PwMS had a relapsing remitting course (89.1%), 9.9% a secondary progressive course, and 1.0% a primary progressive course of the disease. A total of 57 PwMS (57.3%) reported having experienced stigmatization due to MS at least once. Of these, 48 (48.5%) patients experienced public or enacted stigmatization, while 40 (40.8%) experienced self-stigmatization.

In the linear regression model, fatigue (b = -0.199), enacted stigmatization experience (b = -0.627) and SoC (b = 0.654) were significant predictors for the overall scale for QoL.

Fatigue was also a predictor for three of the subscales of QoL (activities of daily living (b = -0.367), psychological well-being (b = -0.176) and symptoms (b = -0.436)). The SoC predicted six subscales of QoL: psychological well-being (b = 0.605), relationships with friends (b = 0.835), relationships with family (b = 0.729), sentimental and sexual life (b = 1.244), coping (b = 0.659) and relationships with the healthcare system (b = 0.571). Enacted stigma predicted three subscales (activities of daily living (b = -1.033), symptoms (b = -1.232) and rejection (b = -1.129) whereas self-stigma only predicted the subscale of rejection (b = -2.301). An overview of all results can be found in Table 2.

**Fig. 1:**
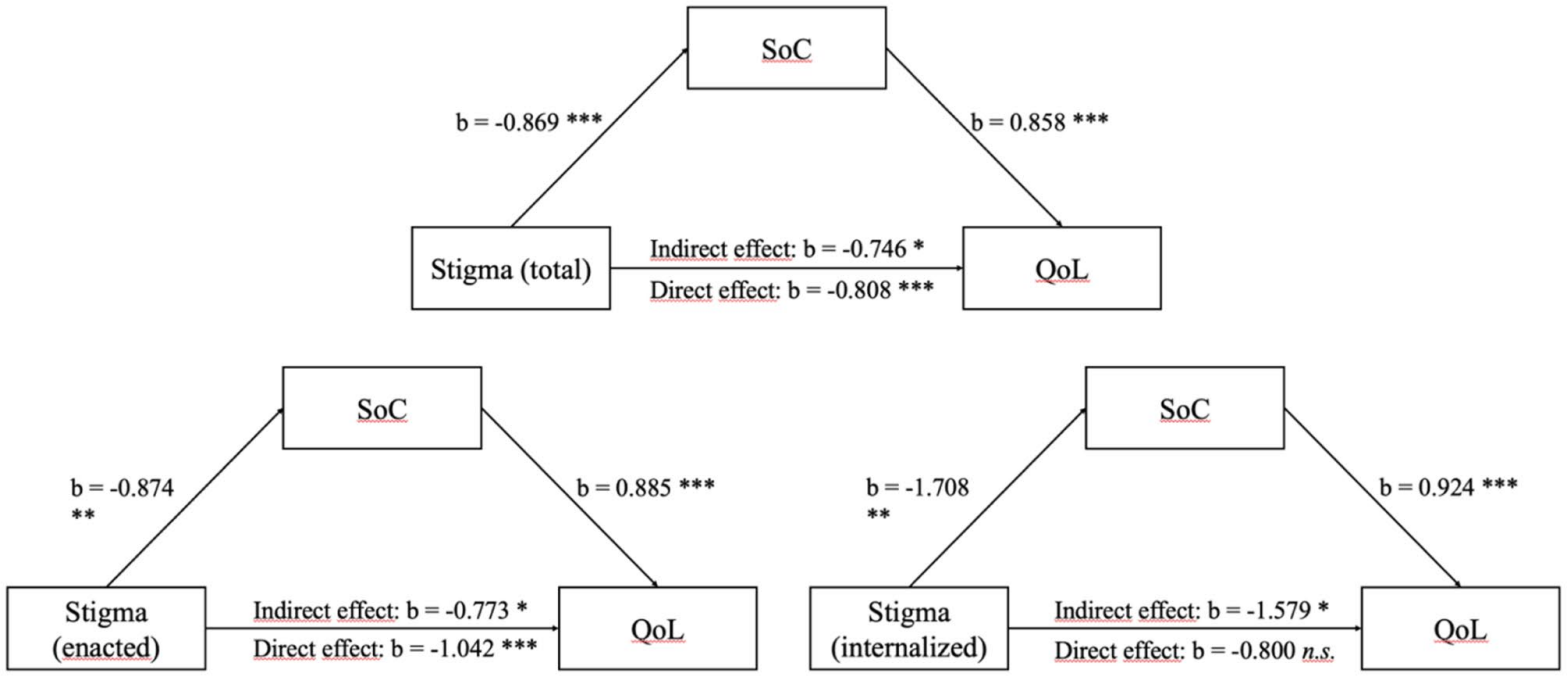
Mediation Analysis

A mediation analysis was performed to examine if the patients’ SoC mediated the association between their stigmatization experiences and their QoL (see Fig. 1). The direct path between stigmatization and QoL remained significant (b = -0.8080, t = -3.95, p < 0.001). However, the indirect effect of both variables also became significant in our mediation model (b = -0.746, CI = -1.174—-0.399). Experiences of stigmatization affected the QoL of PwMS both directly and indirectly, with an indirect effect being mediated through the SoC. Another partial mediation was found when enacted stigmatization was used as the independent variable (b = -0.773, CI = -1.352—-0.339). However, if self- stigma was used as independent variable, the direct path became insignificant and the relationship between self-stigma and QoL was completely mediated by the SoC (b = -1.579, CI = -2.954—-0.669).

*p < .05, **p < .01, ***p < .001, *n.s.* not significant, *SoC* Sense of Coherence, *QoL* Quality of Life

## Discussion

Stigmatization experience affected the QoL in PwMS. This applies both to patients’ overall stigmatization experiences and to enacted and self-stigma, which had various effects on different QoL subscales in our cohort. In general, the participants in our study experienced only a low level of stigmatization. This is consistent with results from previous studies with PwMS, in which the patients also reported a low to moderate overall stigmatization experience [9]. Nevertheless, stigmatization experience were significantly related to QoL. The more stigmatized PwMS felt, the lower was their perceived QoL, which is consistent with the results of Anagnostouli et al. [8]. A more detailed view of the subscales of our QoL questionnaire revealed distinct associations: enacted stigmatization affected the total QoL score as well as the three subscores (activities of daily living, the experience of symptoms and rejection), whereas self-stigma only affected the rejection scale. The different relationships between enacted and self-stigmatization were also investigated by Anagnostouli et al. [8]. In their study, they used a different questionnaire, the Multiple Sclerosis Quality of Life 54, to measure QoL. However, its subdimensions are summarized to either physical or mental health, which at least in part correspond to the dimensions of daily life activities and psychological well-being used in this study [20]. Anagnostouli et al. [8] showed that each dimension of stigmatization correlated negatively with both physical and mental health. They also report a significant impact of enacted stigmatization on physical health and of self-stigmatization on both physical and mental health. Our data is more compatible with that of Broersma et al. [9], according to which enacted stigmatization is predominantly associated with activities of daily living. The subscales activities of daily living and symptoms both refer to patients’ physical limitations. It can be assumed that PwMS with more physical limitations also feel more exposed to negative stereotypes in their social environment due to a more visible impact of MS. On the other hand, the subscales dealing with the relationships with friends, family or the health care system (HCS) seem to be more robust against public stigmatization. The patients apparently have confided in family and friends as well as in the HCS. They do not expect discriminatory treatment by them and therefore do not expect stigmatization. In contrast, the experience of stigmatization had a significant influence on the rejection subscale. It seems clear that PwMS who experienced discrimination feel rejected by their surroundings with a negative effect on their QoL. This assumption is mirrored by the association of self-stigma with the expectation of being rejected.

A significant positive correlation between the SoC and the QoL was found. This result is in line with a large number of studies on the SoC in various chronic diseases, such as those compiled by Eriksson and Lindström in their review on this topic [26]. These results are largely consistent with the results of Broersma et al. [9]. In their study, they showed significant associations between the SoC and all subscales of the QoL. In our study, the SoC had an impact on almost all subscales of the QoL except on the activities of daily living, symptoms and rejection. Interestingly, these are exactly the scales that were influenced by stigma experience.

It seems that the domains affected by enacted stigmatization may be less related to patients' SoC, perhaps because the SoC is not able to buffer the negative impact of discriminatory experiences. Similarly, Broersma et al. [9] were able to show that the SoC is usually only a predictor of QoL if enacted stigmatization was not included. If the patients have already agreed to the negative stereotypes about themselves and thus experienced self-stigma, this can be influenced by the SoC, because of its function as an internal resource.

A high experience of stigmatization was associated with a lower SoC, which in turn was related to lower QoL for PwMS. Conversely, a lower level of stigmatization experience was associated with a higher SoC, which was related to higher QoL. These results are comparable with those of Świtaj [13], who found that SoC acted as a mediator between self-stigmatization and quality of life in patients with mental illness.

In addition to the mediation of stigma experiences via the SoC, the direct path from stigma experience to QoL remained significant. In contrast, the relationship between internalized stigmatization and the QoL was completely mediated by the SoC. A possible explanation would be that experienced discrimination is imposed on patients from their social environment and is related to multiple dimensions of QoL, such as activities of daily living, and are therefore not fully mediated by the SoC. In accordance with this speculation, the relationship between self-stigma and QoL, might be completely mediated by the SoC, since internalized stigma and SoC probably represent partially intersecting cognitive constructs, especially ‘manageability’. As Corrigan et al. [27] have outlined, reduced self-esteem and self-efficacy are immanent consequences of internalized stigma and therefore closely related to the perceived manageability as part of the SoC.

## Limitations

Some limitations concerning this study must be considered. Firs, as in most previous studies, this applies only to the sample. The present study is a cross-sectional sample with a relatively small sample size. However, this is the first study ever regarding stigma in PwMS in Germany. Especially the mediation analysis has never been performed before in this context. It is therefore difficult to draw causal conclusions from the data with absolute certainty. The next step would be to carry out a longitudinal study in which the participants are accompanied over several measurement points. Furthermore, all PwMS were patients of the special outpatient clinic for MS in a German university medical center. This may be an explanation for the rather low experience of stigmatization. PwMS who feel strongly stigmatized tend to avoid medical services [28] and would therefore not attend medical consultations.

A second point is the difficulty of comparing scales used in other studies to measure stigmatization experience and QoL. Many different questionnaires were used across different studies. Additionally, the analyses often focused on only one subscale of either the stigmatization experience or the QoL, so that an exact comparability with the present study was not always given.

## Conclusion

Results from this study show that stigmatization experience and the SoC in PwMS are related with their QoL. Even if the average stigmatization experience was low, the relationship with both QoL and SoC is relevant. In addition, SoC could be found as a mediator between the stigmatization experience and the QoL.

The fact that PwMS are affected by stigmatization has already been shown in previous studies and was confirmed here. On the other hand, SoC and its effects on MS have hardly been investigated so far, and the mediating function of SoC has not been previously demonstrated in any study in PwMS. It provides an indication of the processes by which the stigmatization experience is mediated. This can be a starting point for the development of interventions to improve the QoL in PwMS. To strengthen the SoC in PwMS, both behavioral and perceptual processes should be addressed [29]. This may help PwMS to better manage stigmatization and thus achieve a higher QoL, while a higher SoC can also counteract the development of depression [30].

Since stigmatization affects not only the QoL of PwMS, but also their willingness to seek medical help or to continue to participate in social life, further studies should investigate whether these processes are also mediated through the SoC and if the SoC is affected by anti-stigma interventions. If this were the case, appropriate interventions could be developed or refined to help and support those affected. There is also evidence that the stigmatization experience in PwMS is associated with depression [30] and disability [9]. A link was also found between SoC and fatigue [12] and SoC and depression [31].These individual correlations must be examined more closely in subsequent studies and integrated into an overall model. Not only the direct effects should be measured, but also the possibility of moderation and mediation effects should be considered. In further steps, data from longitudinal studies should be collected to uncover possible causalities.

**Table 1:** Patient characteristics

	n	mean	*SD*	median
Sex (male/female)	101 (74/27)			
Age		46.03	12.21	
Duration		10.72	6.75	
EDSS^a^		2.54	1.88	2.00
BDI^b^		9.11	8.00	
FSMC^c^		57.16	21.83	
FSMC^c^—motor		29.87	11.76	
FSMC^c^—cognitive		27.49	10.92	
SDMT^d^		47.49	13.16	
SOC^e^		50.64	9.28	
Stigma		10.75	3.70	
Stigma—enacted		7.77	2.84	
Stigma—internalized		3.00	1.70	
QoL^f^		79.10	11.76	

*SD* Standard deviation

^a^expanded disability status scale

^b^Beck depression inventory

^c^Fatigue score for motor and cognition

^d^Symbol digit matching task

^e^Sense of Coherence

^f^Quality of Life

**Table 2:** Regression coefficients (and standard errors) showing the predictors of quality of life (QoL) and its subscales in patients with MS

	**QoL – total**	**Activities of daily living**	**Psychological well-being**	**Symptoms**	**Relationships with friends**	**Relationships with family**	**Sentimental and sexual life**	**Coping**	**Rejection**	**Relationships with health care system**
**F**	F (3, 86) = 82,875***	F (4, 85) = 66.789***	F (4, 85) = 32.664***	F (4, 85) = 52.481***	F (1, 88) = 17.191***	F (2, 87) = 8.417***	F (2, 87) = 21.820***	F (2, 87) = 39.120***	F (3, 86) = 14.267***	F (1, 88) = 18.306***
**R**^**2**^	0.743	0.759	0.606	0.712	0.163	0.162	0.334	0.474	0.332	0.172
**Constant**	62.521 (5.367)	119.728 (3.823)	57.635 (11.471)	106.963 (5.124)	26.369 (10.330)	35.236 (11.044)	-13.503 (13.002)	60.133 (12.707)	109.296 (3.882)	92.030 (1.333)
**Gender**	0.059	0.065	**5.795 (2.869)** *	**5.304 (2.364)** *	0.014	0.011	0.093	-0.035	-0.016	-0.005
**Age**	0.029	-0.061	0.059	-0.004	0.119	0.084	-0.066	0.058	0.085	0.071
**Duration**	- 0.062	-0.066	-0.004	-0.073	0.016	0.073	-0.015	-0.005	-0.080	-0.045
**SDMT**^**a**^	0.057	-0.010	-0.031	-0.079	0.015	0.171	**0.460 (0.162)** **	-0.016	-0.056	-0.072
**EDSS**^**b**^	-0.029	**-4.531 (0.602)** ***	-0.051	0.060	0.114	**2.244 (1.043)** *	0.014	-0.101	-0.144	0.092
**BDI**^**c**^	- 0.123	**-0.494 (0.195)** *	**-0.713 (0.271)** *	**-0.449 (0.191)** *	0.073	0.004	-0.063	**-0.940 (0.248)** ***	**-0.422 (0.187)** *	0.176
**FSMC**^**d**^	- 0.199 (0.034) ***	**-0.367 (0.071)** ***	**-0.176 (0.080)** *	**-0.436 (0.067)** **	0.035	-0.103	-0.158	0.034	-0.111	0.003
**Stigma** **enacted**	**-0.627 (0.237)** *	**-1.033 (0.410)** *	0.032	**-1.232 (0.403)** ***	-0.189	-0.003	-0.081	0.029	**-1.129 (0.477)** *	0.111
**Stigma internalized**	-0.030	0.007	-0.134	-0.004	0.104	0.156	0.042	-0.117	**-2.301 (0.829)** **	0.053
**SoC**^**e**^	**0.654 (0.078)** ***	0.107	**0.605 (0.192)** **	-0.034	**0.835 (0.201)** ***	**0.729 (0.209)** **	**1.244 (0.222)** ***	**0.659 (0.216)** **	0.121	**0.571 (0.133)** ***

^a^Symbol digit matching task

^b^expanded disability status scale

^c^Beck depression inventory

^d^Fatigue score for motor and cognition

^e^Sense of Coherence

*p < .05, **p < .01, ***p < .001
